# An interdisciplinary evaluation of community-based TURF-reserves

**DOI:** 10.1371/journal.pone.0221660

**Published:** 2019-08-23

**Authors:** Juan Carlos Villaseñor-Derbez, Eréndira Aceves-Bueno, Stuart Fulton, Alvin Suarez, Arturo Hernández-Velasco, Jorge Torre, Fiorenza Micheli

**Affiliations:** 1 Bren School of Environmental Science & Management, University of California, Santa Barbara, Santa Barbara, CA, United States of America; 2 Nicholas School of the Environment, Duke University, Beaufort, NC, United States of America; 3 Comunidad y Biodiversidad A.C., Guaymas, Sonora, Mexico; 4 Hopkins Marine Station and Stanford Center for Ocean Solutions, Stanford University, Pacific Grove, CA, United States of America; University of Barcelona, SPAIN

## Abstract

Coastal marine ecosystems provide livelihoods for small-scale fishers and coastal communities around the world. Small-scale fisheries face great challenges since they are difficult to monitor, enforce, and manage, which may lead to overexploitation. Combining territorial use rights for fisheries (TURF) with no-take marine reserves to create TURF-reserves can improve the performance of small-scale fisheries by buffering fisheries from environmental variability and management errors, while ensuring that fishers reap the benefits of conservation investments. Since 2012, 18 old and new community-based Mexican TURF-reserves gained legal recognition thanks to a regulation passed in 2012; their effectiveness has not been formally evaluated. We combine causal inference techniques and the Social-Ecological Systems framework to provide a holistic evaluation of community-based TURF-reserves in three coastal communities in Mexico. We find that, overall, reserves have not yet achieved their stated goals of increasing the density of lobster and other benthic invertebrates, nor increasing lobster catches. A lack of clear ecological and socioeconomic effects likely results from a combination of factors. First, some of these reserves might be too young for the effects to show (reserves were 6–10 years old). Second, the reserves are not large enough to protect mobile species, like lobster. Third, variable and extreme oceanographic conditions have impacted harvested populations. Fourth, local fisheries are already well managed, and while reserves may protect populations within its boundaries, it is unlikely that reserves might have a detectable effect in catches. However, even small reserves are expected to provide benefits for sedentary invertebrates over longer time frames, with continued protection. These reserves may provide a foundation for establishing additional, larger marine reserves needed to effectively conserve mobile species.

## Introduction

Marine ecosystems around the world sustain significant impacts due to overfishing and unsustainable fishing practices [1–3]. In particular, small-scale fisheries face great challenges since they tend to be hard to monitor and enforce, given the large number of participants, heterogeneity in fleet, gears, and targeted species; as well as seasonality and geographical distribution [4, 5]. One of the many approaches taken to improve the performance of coastal fisheries and health of the local resources is through the implementation of Territorial Use Rights for Fisheries (TURFs) that contain no-take marine reserves [6–8].

TURFs are a fisheries management tool in which a well-defined group of fishers (*e.g*. fishing cooperatives) have exclusive access to an explicitly delimited portion of the ocean. They promote a sense of stewardship and incentivise resource users to sustainably manage their resources [9–11]. On the other hand, no-take marine reserves (marine reserves from hereinafter) are areas where all extractive activities are off-limits. These can be implemented to protect biodiversity but also as fishery management tools to aid in the recovery of marine stocks. These instruments can be combined by establishing a marine reserve within a TURF, thus making them TURF-reserves [6–8].

Conservation science has shown how well implemented and enforced marine reserves may lead to increased biomass, species richness, and abundance within the protected regions [12, 13], and that these may have a series of additional benefits such as mitigation and adaptation to climate change effects, protection from environmental variability, and fisheries benefits [14–16]. Likewise, research on TURFs has shown that these areas have higher abundance of targeted species than sites operating under open access and even similar to that of marine reserves [9, 17]. The benefits resulting from reserves established within TURFs (*i.e*. TURF-reserves) should be captured exclusively by the group of fishers with exclusive access [7]. Although in theory these systems are expected to be successful [18], there is little empirical evidence of their effectiveness and the drivers of their success.

Recent changes in fisheries regulation in Mexico provide a ripe opportunity to study the effectiveness of community-based TURF-reserves in small-scale fisheries. In Mexico, a legal framework created in 2012 allows fishers to request legal recognition of community-based reserves as “Fish Refuges” (*Zona de Refugio Pesquero*, described in more detail below; [19]). Since 2012, 45 old and new marine reserves have gained legal recognition as Fish Refuges. Of these, 18 were originally implemented within TURFs. However, their effectiveness has not yet been reported in the scientific literature.

Marine reserves have largely been evaluated from an ecological perspective, focusing on ecological indicators such as biomass and species richness or spillover [12, 13, 16]. However, TURF-reserves are intricate social-ecological systems, often implemented with a combination of ecological and social objectives. A customary evaluation of TURF-reserves is unlikely to provide an accurate representation of the changes, benefits, and limitations of such an intervention.

The objective of this work is twofold. First, to provide a holistic evaluation of the effectiveness of community-based TURF-reserves in terms of the changes in biological and socioeconomic indicators and the governance settings under which these develop, which may inform similar processes in other countries. The second objective is to identify opportunities where improvement or adjustment might lead to increased effectiveness of these reserves. We combine causal inference techniques and the Social-Ecological Systems (SES) framework to evaluate community-based TURF-reserves in three coastal communities in Mexico. Our work highlights the benefits of taking an interdisciplinary approach to marine reserve evaluation, and provides a robust evaluation of TURF-reserve effectiveness that may inform adaptive management and future interventions.

## Methods

### TURF-reserves in Mexico

Community-based marine reserves that are implemented within TURFs are a form of TURF-reserve, voluntarily established and enforced by local communities. Community-based spatial closures occur elsewhere, like the *kapu* or *ra’ui* areas in the Pacific Islands [20, 21]. This bottom-up approach can increase compliance and self-enforcement, and reserves can yield benefits similar to systematically-designed reserves [18, 22]. However, community-based reserves can be hard to enforce if they are not legally recognized. In such conditions, TURF fishers must rely on the exclusive access of the TURF to maintain high levels of compliance.

In an effort to bridge this normative gap, Mexican Civil Society Organizations (CSOs) served as a link between fishers and government, and helped create a legal framework that solves this governance issue: Fish Refuges [19]. Fish Refuges can be implemented as permanent, temporary or partial reserves, which can protect one, some, or all resources within their boundaries. One of the ways in which fishing communities have taken advantage of this new tool is by implementing temporary marine reserves within their TURFs with a defined expiration date (often five years). When the expiration date is reached, fishers can chose to open the reserves to fishing or re-establish them. Our work focuses on Fish Refuges implemented as community-based TURF-reserves in small-scale fisheries.

**Fig 1:**
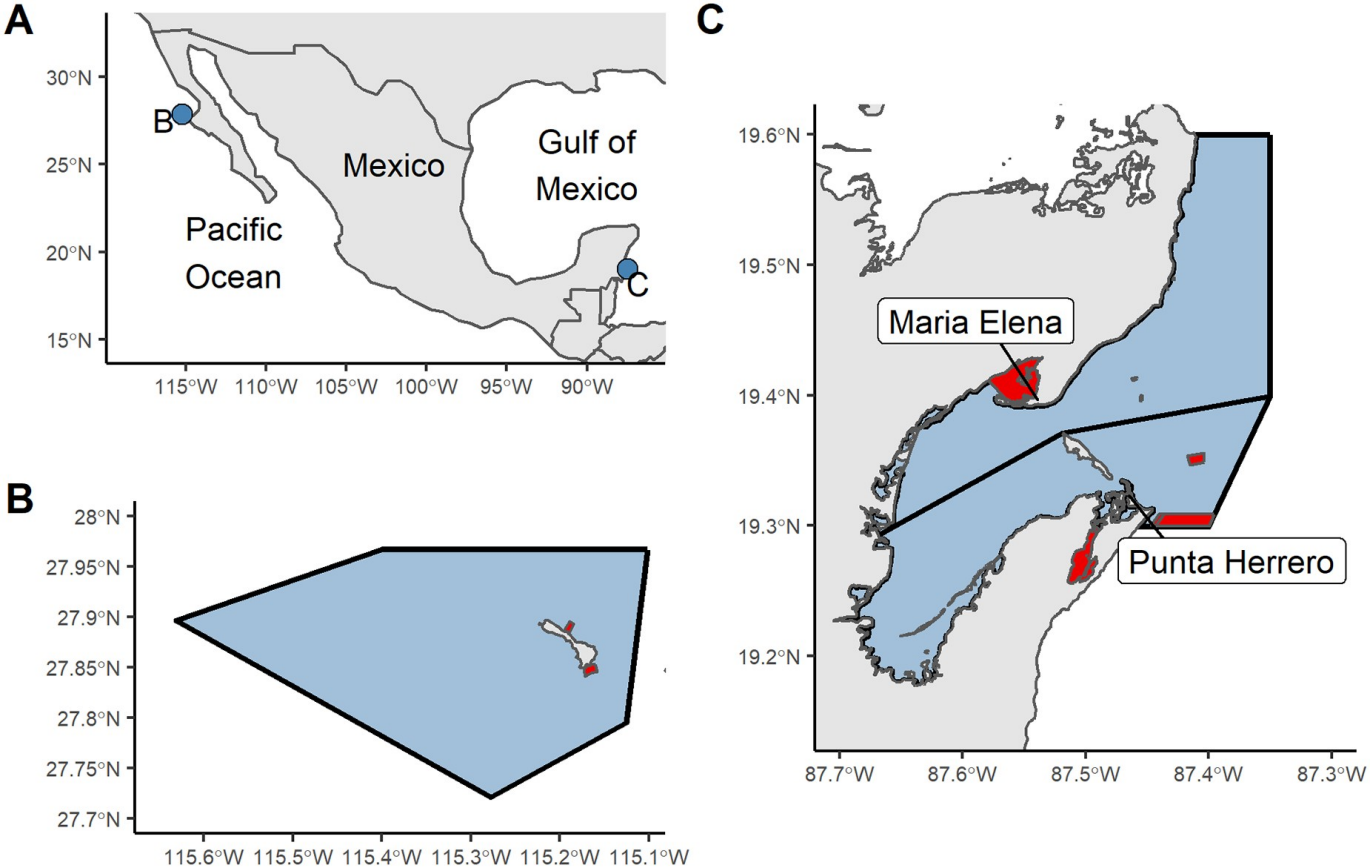
Location of the three coastal communities studied. (A) Isla Natividad (B) is located off the Baja California Peninsula, Maria Elena and Punta Herrero (C) are located in the Yucatan Peninsula. Blue polygons represent the TURFs, and red polygons the marine reserves.

The most common setup of community-based TURF-reserves in Mexico is the following. Fishers from a given community assemble into a fishing cooperative which has exclusive fishing rights over a spatially delimited area (*i.e*. TURFs shown as blue polygons in Fig 1A). Each TURF is exclusively fished by one cooperative, and each community usually hosts no more than one cooperative. The profits from each TURF are shared amongst all fishers from the cooperative [11, 23]. Fishing cooperatives interested in implementing marine reserves within their TURFs (*i.e*. TURF-reserves) work with CSOs to design them. Fishers then ask the government to grant legal recognition to their TURF-reserves as Fish Refuges, as stated in the 2014 regulation [19].

### Study areas

We evaluate three community-based no-take TURF-reserve systems implemented in Mexican TURF-managed fisheries (Fig 1A). The first one was created by the *Buzos y Pescadores de la Baja California* fishing cooperative, located in Isla Natividad in the Baja California Peninsula (Fig 1B). At present, the main fishery in the island is the spiny lobster (*Panulirus interruptus*), but other resources like finfish, sea cucumber, sea urchin, snail, and abalone are also an important source of income. In 2006, the community decided to implement two marine reserves within their fishing grounds. The objective of these reserves was “to protect and recover stocks of commercially important invertebrate species”; mainly lobster and abalone. The reserves were implemented and enforced by the community since 2006, but obtained legal recognition in 2018 [24].

The other two TURF-reserve systems are located in Maria Elena and Punta Herrero, in the Yucatan Peninsula (Fig 1C). In contrast with Isla Nativdad, which hosts a well-established fishing community, Maria Elena is a fishing camp visited intermittently during the fishing season that belongs to the *Cozumel* fishing cooperative. Punta Herrero is home to the *José María Azcorra* fishing cooperative, and similar to Isla Natividad hosts a small community. Their main fishery is the Caribbean spiny lobster (*Panulirus argus*), but they also target finfish in the off-season. Maria Elena and Punta Herrero established eight and four marine reserves in 2012 and 2013, respectively. These reserves have been legally recognized as Fishing Refuges since their original implementation [25, 26] and subsequent re-establishments in 2017 [27].

These communities are representative of their region in terms of ecology, socioeconomic, and governance aspects. Isla Natividad, for example, is part of a greater group of fishing cooperatives belonging to a Federation of Fishing Cooperatives. This group has been identified as a cohesive group that cooperates to better manage their resources [11, 23, 28]. Likewise, Maria Elena and Punta Herrero are representative of fishing cooperatives in the Mexican Caribbean, which are also part of a regional Federation. Together, these three communities provide an accurate representation of other fishing communities that have been historically managed with TURFs in each of their regions. While each region has additional communities that have established community-based TURF-reserves, available data would not allow us to perform the in-depth causal inference analysis that we undertake. Yet, given the similarities among communities and the socioeconomic and governance setting under which they operate, it is safe to cautiously generalize our insights to other similar community-based TURF-reserves in Mexico and elsewhere.

The regulation governing the implementation of Fish Refuges states that these are fishery management tools intended to have conservation and fisheries benefits [19]. For this reason, the main portion of our analyses focuses on a series of biological and socioeconomic indicators that may respond to reserve implementation. However, the effectiveness of conservation and fisheries management interventions also depends on the social and governance structures in place. We therefore incorporate a reduced version of the Social Ecological Systems framework [29] and evaluate variables and indicators known to aid and hinder the effectiveness of management interventions in conservation and fisheries. The incorporation of the SES is not intended to relate different levels of governance with reserve effectiveness, but rather to provide context on the social-ecological system in which reserves develop. The following two sections describe our data collection methods and analyses.

### Data collection

We use three main sources of information to evaluate these reserves across ecological, socioeconomic, and governance dimensions. Biological data come from the annual ecological monitoring of reserve and control sites. Reserve sites are areas where no fishing occurs. Control sites are areas that meet the following criteria: i) habitat characteristics are similar to the corresponding reserves, ii) presumably had a similar probability of being selected as reserves during the design phase, iii) are located within the TURF, where fishing occurs, and iv) are not directly adjacent to the reserves to avoid confounding due to spillover effect (sites were at least 1 km apart). We focus our evaluation on sites where data are available for reserve and control sites, before and after the implementation of the reserve. This provides us with a Before-After-Control-Impact (*i.e*. BACI) sampling design that allows us to capture and control for temporal and spatial dynamics and causally attribute the changes to the reserve [30–34].

The biological data are collected by members from each community and personnel from the Mexican CSO *Comunidad y Biodiversidad* (COBI). Trained divers record species richness and abundances of fish and invertebrate species along replicate transects (30 × 2 m each) at depths 5-20 m in the reserves and control sites [35–37]. Size structures are also collected during fish surveys, where divers estimate fish length to the nearest centimeter. All sites were surveyed annually, and at least once before implementation of the reserves. A summary of sampling effort and time series, as well as species checklist, are shown in the supplementary materials (Tables A-B, Figs A-D, and Tables I-J in S1 Text).

Socioeconomic data contain monthly lobster landings (Kg) and revenues (in Mexican Pesos; MXP) for TURF-managed cooperatives with and without marine reserves. These were requested from the National Commission for Aquaculture and Fisheries (*Comisión Nacional de Acuacultura y Pesca*; CONAPESCA) via the access to information act. In this case our treated unit are the cooperatives (*i.e*. communities) that have implemented a reserve within their TURF, and the controls are adjacent communities that have a TURF but did not implement a reserve. Cooperatives incorporated in this analysis have similar number of members, belong to larger regional-level Cooperative Federations, and are exposed to the same markets and institutional frameworks, making them plausible controls [11, 23, 38]. Landings and revenues were aggregated at the cooperative-year level, and revenues were adjusted to represent 2014 values by the Consumer Price Index for Mexico [39]. A table with summary statistics and time series for this data are provided in the supplementary materials (Table C and Fig E in S1 Text).

Data for the evaluation of the SES were collected at the community-level, and focused on the Resource Systems, Resource Units, Actors, and Governance System (Table 1). Data come from official documents used in the design, creation, and implementation of the marine reserves. These include the technical studies that the cooperatives submit when they request recognition of their reserves, as well as the official enactments [24–26]. We also complimented information based on the authors’ experience and knowledge of the communities (RS5, RU5, A3, GS6.2, GS99.1, GS9.2, and GS10.1). In Table 1, the alphanumeric codes follow [40], and an asterisk denotes variables incorporated based on [41] and [42].

**Table 1:** Variables for the social-ecological system analysis.

Variable	Narrative
**Resource System (RS)**	
RS2—Clarity of system boundaries: Clarity of geographical boundaries of TURF and reserves	Individual TURF and reserve boundaries are explicitly outlined in official documents that include maps and coordinates. Reserve placement is decided by the community. Fishers use GPS units and landmarks.
RS3—Size of resource system: TURF Area (Km^2^)	IN = 889.5; ME = 353.1; PH = 299.7
RS3—Size of resource system: Reserve area (Evaluated reserve area; Km^2^)	IN = 2 (1.3); ME = 10.48(0.09); PH = 11.25 (4.37)
RS4.1—Stock status: Status of the main fishery	Lobster stocks are well managed, and are (IN) or have been (ME, PH) certified by the Marine Stewardship Council.
*RS5—Age of reserves: Years since reserves were implemented	IN = 12; ME = 6; PH = 5
**Resource Unit (RU)**	
RU1—Resource unit mobility	Adult spiny lobsters can move between 1 and 10 Km, while larvae can have displacements in the order of hundreds of Km (Aceves-Bueno et al., 2017; Green et al., 2017).
RU5—Number of units (catch diversity): Number of targeted species	Lobster is their main fishery of these three communities, but they also target finfish (2 spp each). Additionally, fishers from Isla Natividad target other sedentary benthic invertebrates (4 spp).
**Actors (A)**	
A1—Number of relevant actors: Number of fishers	IN = 98; ME = 80; PH = 21
*A3—Isolation: Level of isolation of the fishing grounds	Their fishing grounds and reserves are highly isolated and away from dense urban centers. IN lies 545 Km south from Tijuana, and ME and PH 230 Km south from Cancun, where the nearest international airports are located.
**Governance system (G)**	
GS6.1.4.3—Territorial use communal rights: Presence of institutions that grant exclusive harvesting rights	Each community has exclusive access to harvest benthic resources, including lobster. These take the form of Territorial User Rights for Fisheries granted by the government to fishing cooperatives.
GS6.2—Operational rules: Rules implemented by individuals authorized to partake on collective activities	Fishers have rules in addition to what the legislation mandates. These are: larger minimum catch sizes, lower quotas, and assigning fishers to specific fishing grounds within their TURF.
GS9.1—Social monitoring: Monitoring of the activities performed by cooperative members and external fishers	Fishing cooperatives have a group (Consejo de vigilancia) that monitors and enforces formal and internal rules. They ensure fishers of their fishing cooperative adhere to the established rules, and that foreign vessels do not poach their TURF and reserves.
GS9.2—Biophysical monitoring: Monitoring of biological resources, including targeted species	Fishers perform annual standardized underwater surveys in the reserves and fishing grounds. Recently, they have installed oceanographic sensors to monitor oceanographic variables.
GS10.1—Graduated sanctions	Fishers have penalties for breaking collective-choice rules or fishing inside the reserves. These may range from scoldings and warnings to not being allowed to harvest a particular resource or being expelled from the cooperative.

IN = Isla Natividad, ME = Maria Elena, PH = Punta Herrero. The presented narrative applies equally for all communities unless otherwise noted. An asterisk (*) denotes variables incorporated into the framework.

### Data analysis

We evaluate the effect that the TURF-reserves have had on four ecological and two socioeconomic indicators shown in Table 2. Recall that reserves were implemented to protect lobster and other benthic invertebrates. However, we also use the available fish and invertebrate data to test for associated co-benefits.

**Table 2:** List of indicators used to evaluate the effectiveness of marine reserves, grouped by category.

Indicator	Units
**Biological**	
Lobster density	org m^−2^
Invertebrate density	org m^−2^
Fish density	org m^−2^
Fish biomass	Kg m^−2^
**Socioeconomic**	
Income from target species	M MXP
Landings from target species	Metric Tonnes

We use a difference-in-differences analysis to evaluate these indicators. This approach is widely used in econometric literature to estimate the average treatment effect of an intervention, like the impact of minimum wage increases on employment rates [43]. In our case it allows us to estimate the effect that the reserve had on each biological and socioeconomic indicator (Table 2) by comparing trends across time and treatments since reserve implementation [33, 34, 44]. To perform difference-in-differences, we regress the indicator of interest on a dummy variable for treatment, a dummy variable for years, and the interaction term between these with a multiple linear regression of the form:

$$I_{i,t}=\alpha +\gamma_{t}Year_{t}+\beta Zone_{i}+\lambda_{t}Year_{t}\times Zone_{i}+\epsilon_{i,t}$$ (1)

Where year-level fixed effects capturing a temporal trend are represented by *γ*_*t*_*Year*_*t*_, and *βZone*_*i*_ captures the difference between reserve (*Zone* = 1) and control (*Zone* = 0) sites. The effect of the reserve is captured by the λ_*t*_ vector of coefficients, and represents the difference observed between the control site before the implementation of the reserve and the treated sites at time *t* after controlling for other time and space variations (*i.e*. *γ*_*t*_ and *β* respectively). Therefore, we would expect this term to be positive if the indicator increases because of the reserve. Finally, *ϵ*_*i*,*t*_ represents the error term of the regression.

Socioeconomic indicators are evaluated with a similar approach. Due to data constrains, we only evaluate socioeconomic data for Isla Natividad (2000–2014) and Maria Elena (2006–2013). Neighboring communities are used as counterfactuals that allow us to control for unobserved time-invariants. Each focal community (*i.e*. Isla Natividad and Maria Elena) has three counterfactual communities.

$$I_{i,t}=\alpha +\gamma_{t}Year_{t}+\beta Treated_{i}+\lambda_{t}Year_{t}\times Treated_{i}+\epsilon_{i,t}$$ (2)

The coefficient interpretations remains as for Eq 1, but in this case the *Treated* dummy variable indicates if the community has a reserve (*Treated* = 1) or not (*Treated* = 0).

These regression models allow us to establish a causal link between the implementation of marine reserves and the observed trends by accounting for temporal and site-specific dynamics [32]. Since we are interested in the effectiveness of each reserve system, we fit one model for each indicator in each community (*e.g*. there are three models for lobster density, one for each community). This gives us a total of 12 biological model fits and four socioeconomic model fits. Model coefficients were estimated via ordinary least-squares and used heteroskedastic-robust standard error correction [45]. All analyses were performed in R 3.5.2 and R Studio version 1.1.456 [46]. All data and code needed to reproduce our analyses are available in a GitHub repository at: https://github.com/jcvdav/ReserveEffect.

TURF-reserve systems are inherently intricate social-ecological systems, and their effectiveness must depend on how environmental and social factors combine and interact [7, 29]. When evaluating the effects of TURF-reserves, it is important to consider not only the indicators of interest, but also the governance settings under which a reserve operates. We use the SES framework to qualitatively evaluate each community and create a narrative that provides context for each of them. The use of this framework standardizes our analysis and allows us to communicate our results in a common language across fields by using a set of previously defined variables and indicators. Due to the lack of sufficient information to quantitatively operationalize the social-ecological systems framework for these case studies (as done in [47]), we followed a similar approach to [40], who used the SES framework as a classification system of the available information to qualitatively analyze fisheries systems. We based our variable selection primarily on previous analyses of Mexican fishing cooperatives [40, 47]. We also incorporate other relevant variables known to influence reserve performance, such as age and size of reserve or isolation of the system [41, 42]. Table 1 shows the selected variables, along with definitions and values.

## Results

The following sections present the effect that marine reserves had on the biological and socioeconomic indicators for each coastal community. Results are presented in terms of difference through time and across sites, relative to the control site on the year of implementation (*i.e*. the difference-in-differences estimate or effect size λ_*t*_ from Eqs 1 and 2). We also provide an overview of the governance settings of each community, and discuss how these might be related to the effectiveness and performance of the reserves.

### Biological effects

**Fig 2:**
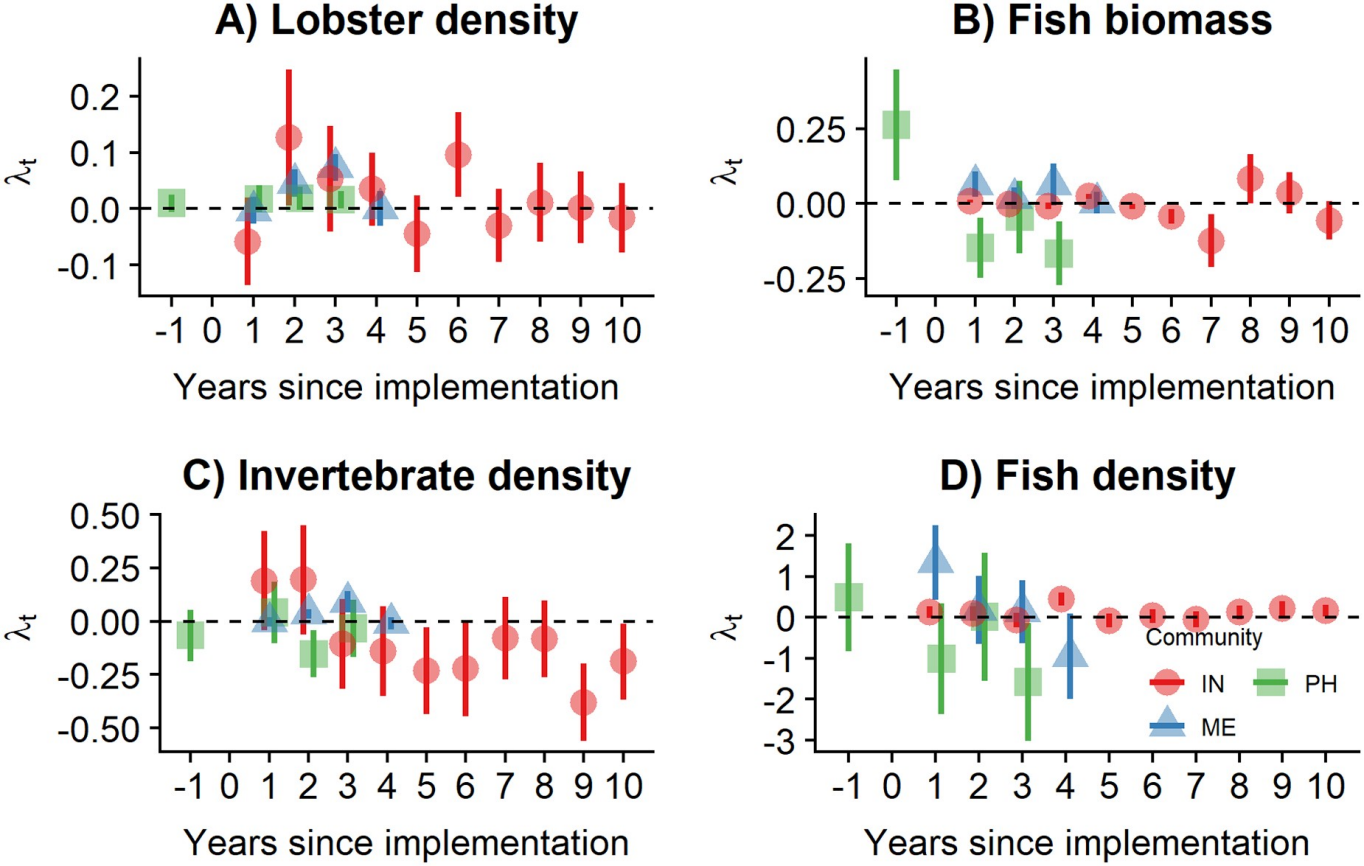
Effect sizes for biological indicators. Points indicate the effect size and error bars are heteroskedastic-robust standard errors. Years have been centered to year of implementation. Colors and shapes denote communities: Isla Natividad (IN; red circles), Maria Elena (ME; blue triangles), and Punta Herrero (PH; green squares). Points are jittered hotizontally to avoid overplotting.

Indicators showed ambiguous responses through time for each reserve. Fig 2A shows positive effect sizes for lobster densities in Isla Natividad and Punta Herrero during the first years, but the effect is eroded through time. In the case of Maria Elena, positive changes were observed in the second and third years. These effects were in the order of 0.01 extra organisms m^−2^, but were only significantly different from zero for Maria Elena (*p* < 0.05). Likewise, no significant changes were detected in fish biomass or invertebrate and fish densities (Fig 2B–2D). Invertebrate and fish densities showed positive trends in all reserves for some years. However, these were not statistically significant. Figures with time series of indicators and tables with model coefficients and main effects are presented in the supplementary materials (Figs A-D and Tables D-F in S1 Text).

### Socioeconomic effects

**Fig 3:**
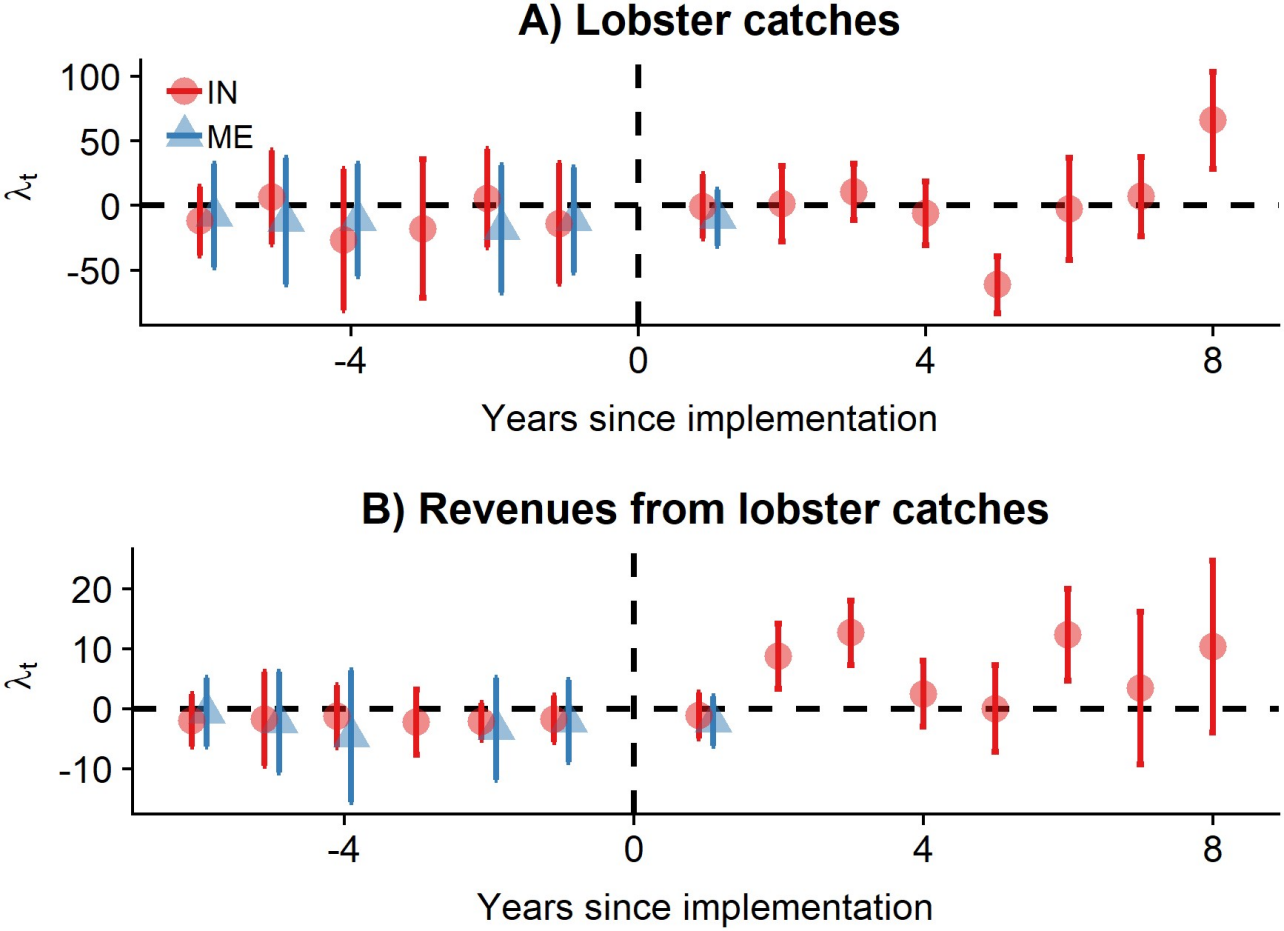
Effect sizes for socioeconomic indicators. Points indicate the effect size and error bars are heteroskedastic-robust standard errors for lobster catches and revenues at Isla Natividad (IN; red circles) and Maria Elena (ME; blue triangles). Years have been centered to year of implementation. Points are jittered hotizontally to avoid overplotting.

Lobster landings and revenue were only available for Isla Natividad and Maria Elena (Fig 3). For all years before implementation, the effect sizes are close to zero, indicating that the control and treatment sites have similar pre-treatment trends, suggesting that these are plausible controls. However, effect sizes do not change after the implementation of the reserve. Interestingly, the negative effect observed for Isla Natividad on year 5 corresponds to the 2011 hypoxia events [15]. The only positive change observed in lobster landings is for Isla Natividad in 2014 (*p* < 0.1). The year of post-implementation data for Maria Elena does not show a significant effect of the reserve. Isla Natividad shows higher revenues after the implementation of the reserve, as compared to the control communities, though these changes are only significant for the third year (*p* < 0.05). Full tables with model coefficients are presented in the supplementary materials (Tables G-H in S1 Text).

### Governance

We find that the analyzed communities share similarities known to foster sustainable resource management and increase reserve effectiveness. For example, fishers operate within clearly outlined TURFs (RS2, GS6.1.4.3) that provide exclusive access to resources and reserves. Along with their relatively small groups (A1—Number of relevant actors), Isolation (A3), Operational rules (GS6.2), Social monitoring (GS9.1), and Graduated sanctions (GS10.1), these fisheries have strong governance structures that enable them to monitor their resources and enforce rules to ensure sustainable management [11, 38].

In general, success of conservation initiatives depends on the incentives of local communities to maintain a healthy status of the resources upon which they depend [48]. Due to the clarity of access rights and isolation, the benefits of conservation directly benefit the members of the fishing cooperatives, which have favored the development of efficient community-based enforcement systems. However, our SES analysis also highlights factors that might hinder reserve performance or mask outcomes. While total reserve size ranges from 0.2% to 3.7% of the TURF area, individual reserves are often small (RS3); the largest reserve is only 4.37 km^2^, and the smallest one is 0.09 km^2^. Reserves are also relatively young (RS5). Additionally, fishers harvest healthy stocks (RS4.1), and it is unlikely that marine reserves will result in increased catches.

## Discussion

Our results indicate that these TURF-reserves have not increased lobster densities. Additionally, no co-benefits were identified when using other ecological indicators aside from the previously reported buffering effect that reserves can have to environmental variability in Isla Natividad, as well as positive trends in invertebrate and fish densities [15, 34]. The socioeconomic indicators pertaining landings and revenues showed little to no change after reserve implementation. Our qualitative implementation of the social-ecological systems framework allowed us to systematically identify important differences between the case studies’ governance systems and incorporate other characteristics of these fisheries neglected during the process of data collection (Table 1). These communities exhibit all the social enabling conditions for effective reserve and resource management. Here we discuss possible shortcomings in our analyses as well possible explanations for the lack of effectiveness.

While many ecology studies have used BACI sampling designs and respective analyses (*e.g*. [30]), few conservation studies have done so to evaluate the effect of an intervention (*e.g*. [13, 31, 33, 44]) which has resulted in a call for more robust analyses in conservation science [49, 50]. Our approach to evaluate the temporal and spatial changes provides a more robust measure of reserve effectiveness, and captures previously described patterns. For example, the rapid increase observed for lobster densities in Isla Natividad on the sixth year (*i.e*. 2012; Fig 2A), occurs a year after the hypoxia events described by [15], which caused mass mortality of sedentary organisms such as abalone and sea urchins, but not lobster and finfish.

While the use of causal inference techniques may help us support evidence-based conservation, spatial connectivity between reserve and control sites, stockpiling, and backstopping may confound the results [33]. Given that we find no clear evidence of reserve effectiveness, one might say that our reserve and control sites are not spatially independent. This would imply that the recovery within the reserve quickly results in recovery outside the protected area. However, indicators show little to no temporal variation (Figs A-D in S1 Text), and it is unlikely that this effect would be observed under current reserve designs, as detailed below.

Our analyses of socioeconomic indicators has three limitations. First, we only look at landings and revenues by landings for communities with and without TURF-reserves. There are a number of other possible indicators that could show a change due to the implementation of the reserve. Notably, one often cited in the literature is additional benefits, such as tourism [51]. However, it is unlikely that the evaluated communities will experience tourism benefits due to their remoteness and the lack of proper infrastructure to sustain tourism. A second limitation of our socioeconomic analysis is that we do not observe effort data, which may mask the effect of the reserve. For example, if catches remain relatively unchanged but fishing effort decreased, that would imply a larger catch per unit effort and thus higher profitability, provided that cost per unit effort does not increase. Likewise, it is possible that fishing effort increased around reserves to maintain the historical levels of landings. A final limitation applies to Maria Elena, where we only observe landings and income for one year after reserve implementation. While one would not expect to observe increased landings or income in such a short period, a spatial closure might cause total catches to decline, especially if effort is held constant.

Lack of evidence of reserve effectiveness is commonly associated to lack of proper enforcement and compliance, as well as capacity shortfalls [41, 42, 52]. However, it is unlikely that the evaluated reserves are subject to these common problems. The reserves are implemented within TURFs, which provide a sense of ownership of resources and promotes sustainable management [23]. The same incentives that ensure sustainable management in a TURF should extend to the reserves that these contain. This is supported by our SES analysis, which suggests that these communities exhibit strong governance and enforcement structures. Instead, the lack of effectiveness observed may be driven by a combination of factors, such as age of the reserves, sub-optimal reserve design, or environmental variation. The next paragraphs provide a detailed discussion of these possible causes.

A first possible explanation for the lack of effectiveness may be the young age of the reserves. Literature shows that age and enforcement are important factors that influence reserve effectiveness [42, 53]. Isla Natividad has the oldest reserves, and our SES analysis suggests that all communities have a well-established community-based enforcement system. With these characteristics, one would expect the reserves to be effective. In fact, the oldest reserves show some positive trends for invertebrate and fish densities, as well as income. Maria Elena and Punta Herrero are relatively young reserves (*i.e*. <6 years old; RS5 in Table 1) and effects may not yet be evident; community-based marine reserves in tropical ecosystems may take six years or more to show a spillover effect [54].

Another key condition for effectiveness is reserve size [42], and the lack of effectiveness can perhaps be attributed to poor ecological coherence in reserve design (*sensu* [55]). In Isla Natividad, the reserves can yield fishery benefits for the abalone fishery [56], however, abalone are less mobile than lobsters, and perhaps the reserves provide enough protection to these sedentary molusks, but not lobsters. Small reserves in the Mediterranean Sea have shown that the effect of a reserve is only observable for species with a home range smaller than the reserve [57]. However, design principles developed for marine reserves in the Caribbean state that reserves “should be more than twice the size of the home range of adults and juveniles”, and suggest that reserves seeking to protect spiny lobsters should have at least 14 km across [58]. As shown in the SES analysis, the size of the marine reserves appears small compared to the movement capacity of the main targeted species (RU1, RS3; Table 1). Furthermore, fishers may favor implementation of reserves that pose low fishing costs due to their small size or location. Our analysis of economic data supports this hypothesis, as neither landings nor revenues showed the expected short-term reductions associated to the first years of reserve implementation [59].

Even if reserves had appropriate sizes and were placed in optimal locations, there are other plausible explanations for the observed patterns. For instance, marine reserves are only likely to provide fisheries benefits if initial population sizes are low and the fishery is poorly managed [60, 61]. Both lobster fisheries were certified by the Marine Stewardship Council and are managed via species-specific minimum catch sizes, seasonal closures, protection of “berried” females, and escapement windows where traps are allowed [62]. It is uncertain whether such a well-managed fishery will experience additional benefits from small marine reserves; reserves implemented in TURFs where fishing pressure is already optimally managed will still show a trade-off between fisheries and conservation objectives [8]. In contrast, invertebrate fisheries have seen declining catches and finfish fisheries are not managed under TURFs, which may explain the positive trends observed. Furthermore, TURFs alone can have greater biomass and richness than areas operating under open access [9]. This might reduce the difference between indicators from the TURF and reserve sites, making it difficult to detect such a small change. Further research should focus on evaluating sites in the reserve, TURF, and open access areas or similar Fish Refuges established without the presence of TURFs where the impact of the reserves might be greater.

Finally, extreme conditions, including prolonged hypoxia, heat waves, and storms have affected both the Pacific and Caribbean regions, with large negative impacts on coastal marine species and ecosystems [63–65]. The coastal ecosystems where these reserves are located have been profoundly affected by these events [15, 66]. Effects of protection might be eliminated by the mortalities associated with these extreme conditions.

While the evaluated reserves have failed to provide clear fishery benefits to date, there are a number of additional ecological, fisheries, and social benefits. Marine reserves provide protection to a wider range of species and vulnerable habitat. Previous research focusing on these specific sites has shown that they serve as an insurance mechanism against uncertainty and errors in fisheries management, as well as mild environmental shocks [14, 15, 67, 68]. Self-regulation of fishing effort can serve as a way to compensate for future declines associated to environmental variation [69]. Furthermore, embarking on a marine conservation project can bring the community together, which promotes social cohesion and builds social capital [37]. Showing commitment to marine conservation and sustainable fishing practices has allowed fishers to have greater bargaining power and leverage over fisheries management [70]. These additional benefits might explain why communities show a positive perception about their performance and continue to support their presence by re-establishing the reserves [38, 71].

In terms of fisheries regulation in Mexico, our work only evaluates Fish Refuges established within TURFs. Future research should aim at evaluating other Fish Refuges established as bottom-up processes but without the presence of TURFs (*e.g*. [72]), others established through top-down processes (*i.e*. Ref. [73]), as well as the relationship between governance and effectiveness across this gradient of approaches.

## Conclusion

Our objectives were to evaluate the effectiveness of community-based marine reserves implemented under a new regulation, and learning from this process to inform management of these reserves as well as similar processes elsewhere. We do not find clear evidence of an increase in lobster densities or catches, and implementation of reserves did not come at a cost (*i.e*. reduction in catches or revenues). After identifying this lack of effectiveness, we used the SES framework to look for alternative explanations. The communities seem to exhibit all social and governance conditions that would result in reserve effectiveness. However, these reserves may not be large enough to provide the necessary protection that would lead to increases in biomass. For the particular case of the reserves that we evaluate, the possibility of expanding reserves or merging existing polygons into larger areas should be evaluated and proposed to the communities.

With other projects in mind, bottom-up design and implementation processes like the ones in the evaluated reserves must be promoted. However, these should not come at the cost of setting design principles aside. Having full community support surely represents an advantage, but it is important that community-based TURF-reserves meet essential design principles such as size and placement so as to maximize their effectiveness. Furthermore, conservation and advocacy groups should consider the opportunity costs of such interventions (*sensu* [74]) and evaluate the potential of other approaches and alternative investments that may yield similar benefits.

## Supporting information

S1 Text Supplementary text. Additional figures and tables with summary information. Table A with invertebrate sampling effort. Table B with fish sampling effort. Table C with summary of socioeconomic data. Table D with coefficient estimates for biological indicators in Isla Natividad. Table E with coefficient estimates for biological indicators in Maria Elena. Table F with coefficient estimates for biological indicators in Punta Herrero. Table G with coefficient estimates of socioeconomic indicators in Isla Natividad. Table H with coefficient estimates of socioeconomic indicators in Maria Elena. Fig A with time series of mean annual lobster density for each community. Fig B with mean annual invertebrate densities for each community. Fig C with mean annual fish biomass for each community. Fig D with mean annual fish density for each community. Fig E with time series of socioeconomic indicators. Table I with a checklist of invertebrate species. Table J with a checklist of fish species.
(PDF)
