# Supplementary material for: An interdisciplinary evaluation of community-based TURF-reserves
Source: PLoS One. 2019 Aug 23;14(8):e0221660. doi: 10.1371/journal.pone.0221660 (PMC6707568; doi:10.1371/journal.pone.0221660)
Supplement: S1 Text — Additional figures and tables with summary information. Table A with invertebrate sampling effort. Table B with fish sampling effort. Table C with summary of socioeconomic data. Table D with coefficient estimates for biological indicators in Isla Natividad. Table E with coefficient estimates for biological indicators in Maria Elena. Table F with coefficient estimates for biological indicators in Punta Herrero. Table G with coefficient estimates of socioeconomic indicators in Isla Natividad. Table H with coefficient estimates of socioeconomic indicators in Maria Elena. Fig A with time series of mean annual lobster density for each community. Fig B with mean annual invertebrate densities for each community. Fig C with mean annual fish biomass for each community. Fig D with mean annual fish density for each community. Fig E with time series of socioeconomic indicators. Table I with a checklist of invertebrate species. Table J with a checklist of fish species. (PDF) [file pone.0221660.s001.pdf]

Supporting Information for “An interdisciplinary evaluation of community-based TURF-reserves”

Table A. Invertebrate sampling effort. Number of invertebrate transects performed in each site of each community.

Community	Control	Reserve	Years of monitoring
Isla Natividad	415	244	10
Maria Elena	27	21	4
Punta Herrero	51	78	4

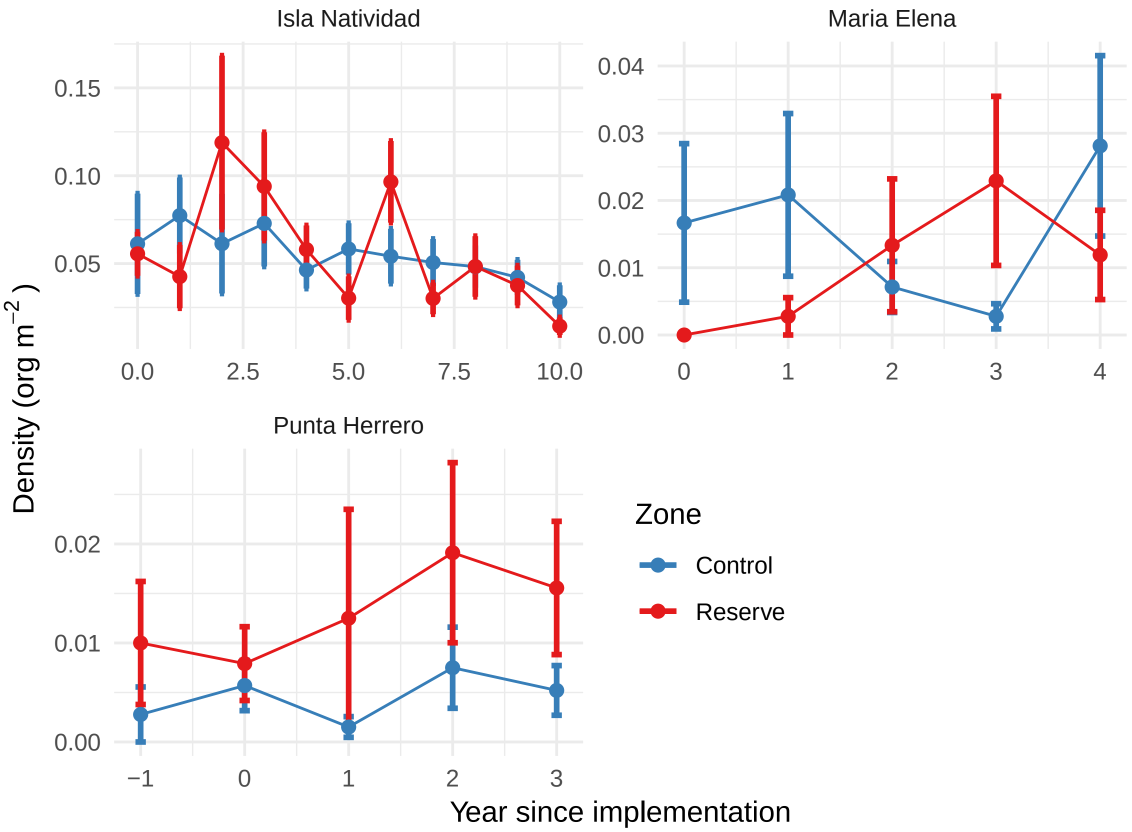

Figure A. Mean annual lobster density. Dots indicate mean, bars indicate standard errors.

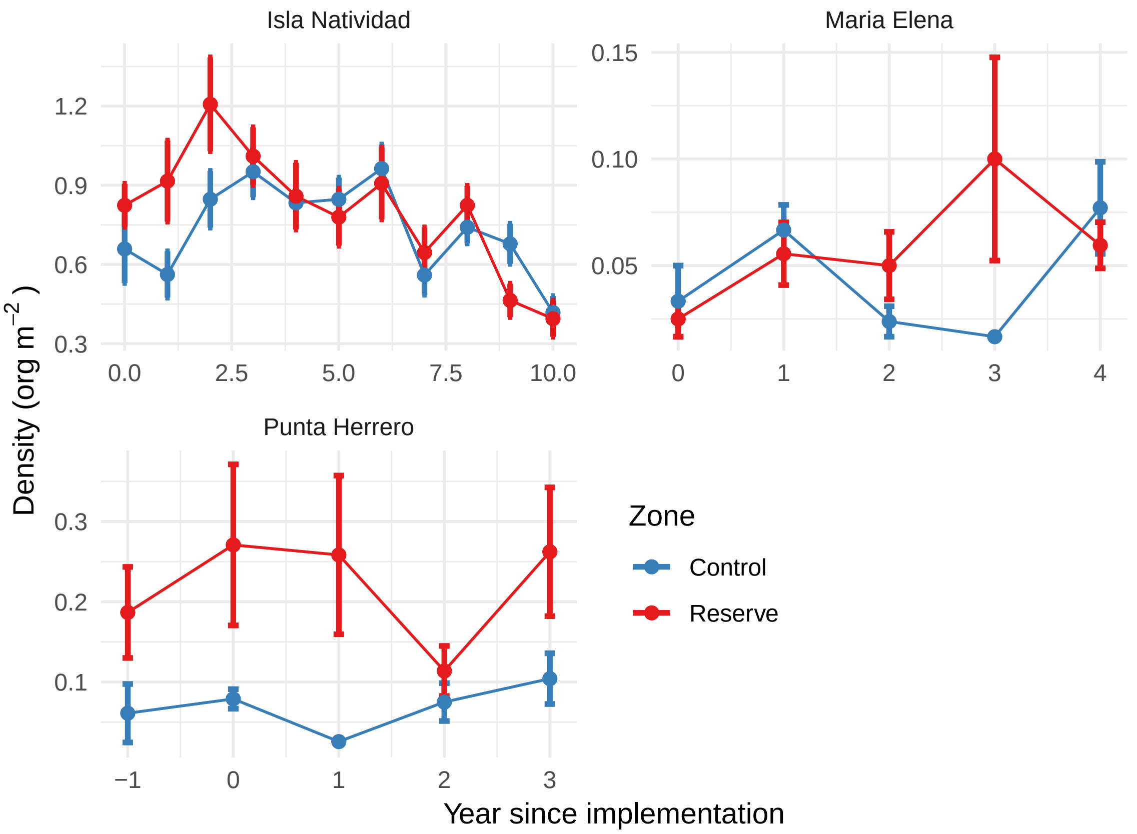

Figure B. Mean annual invertebrate density. Dots indicate mean, bars indicate standard errors.

Table B. Fish sampling effort. Number of invertebrate transects performed in each site of each community.

Community	Control	Reserve	Years of monitoring
Isla Natividad	400	241	10
Maria Elena	44	45	4
Punta Herrero	82	85	4

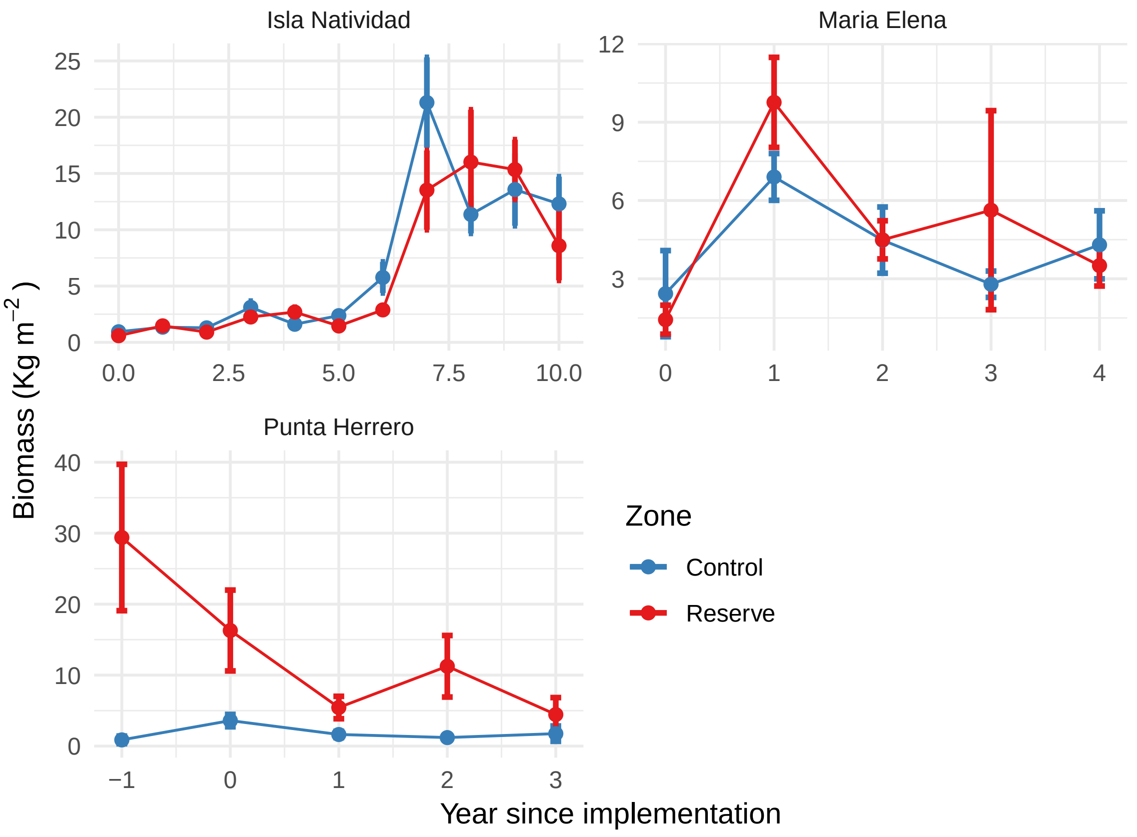

Figure C. Mean annual fish biomass Dots indicate mean, bars indicate standard errors.

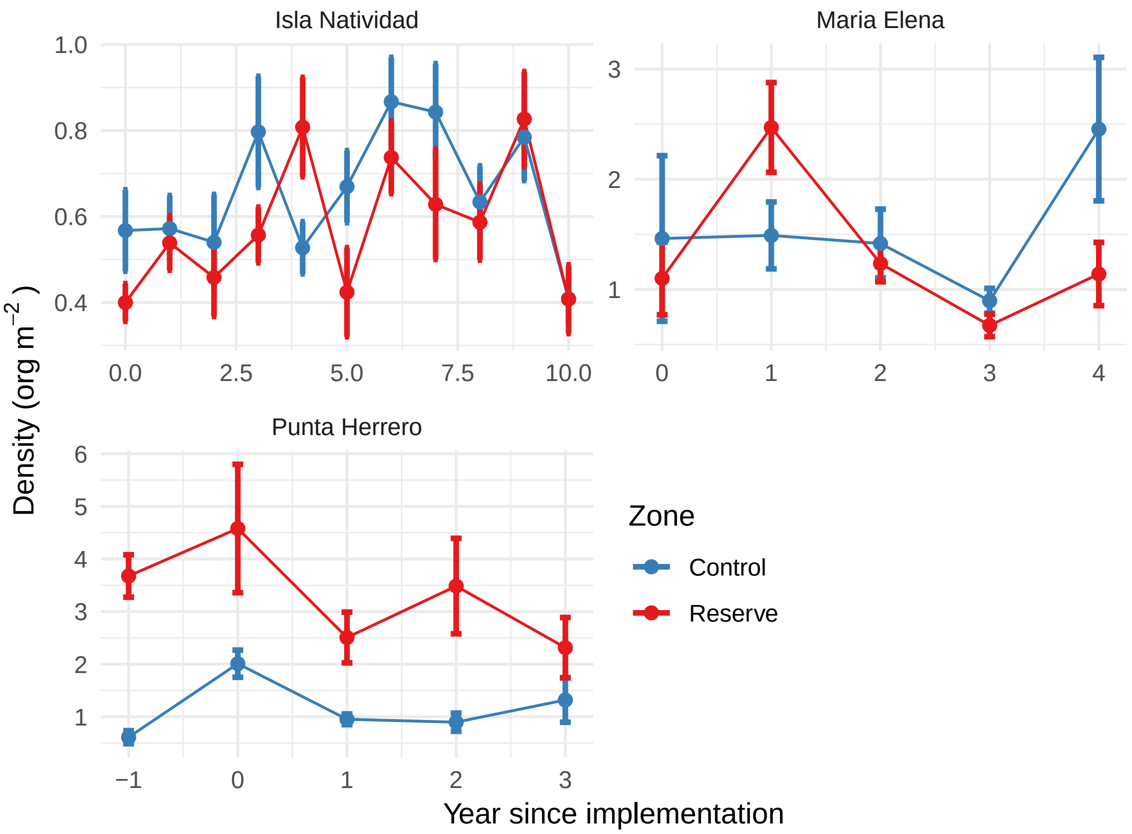

Figure D. Mean annual fish density Dots indicate mean, bars indicate standard errors.

Table C. Summary of socioeconomic data. Mean ex-vessel prices (MXP / Kg) for lobster (*Panulirus spp.*) on each TURF (community) and group (Control = only TURF, Treated = TURF and reserve).

Community	Group	TURF	Before	After
Yucatan Peninsula	Control	Langosteros Del Caribe	165.58	176.72
Yucatan Peninsula	Control	Vigia Chico	177.66	175.31
Yucatan Peninsula	Treated	Cozumel	178.21	170.49
Baja California	Control	Bahia Tortugas	155.60	122.46
Baja California	Control	La Purisima	67.54	157.88
Baja California	Control	Pesc Nacionales De Abulon	67.16	88.79
Baja California	Treated	Buzos Y Pescadores De La Baja California	89.35	190.07

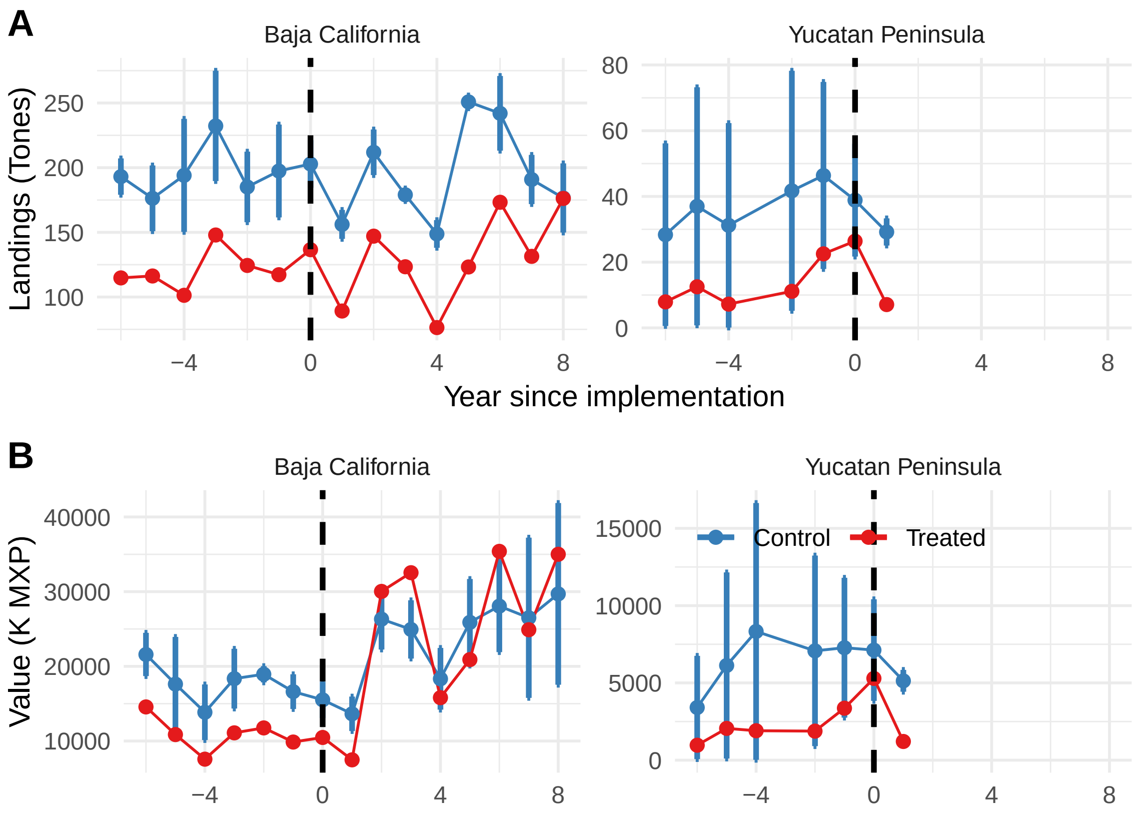

Figure E. Time series of socioeconomic indicators. A) Mean annual value of landings and B) Mean landings.

Table D. Coefficient estimates of biological indicators for Isla Natividad.

	Dependent variable:			
	Lobster density	Fish biomass	Invertebrate density	Fish density
	(1)	(2)	(3)	(4)
zonaReserva	−0.011 (0.058)	−0.006* (0.003)	0.165 (0.152)	−0.167 (0.104)
year1	0.032 (0.067)	0.007 (0.004)	−0.097 (0.155)	0.005 (0.122)
year2	0.0002 (0.077)	0.006 (0.005)	0.188 (0.167)	−0.027 (0.146)
year3	0.023 (0.072)	0.036*** (0.010)	0.293* (0.161)	0.230 (0.160)
year4	−0.030 (0.058)	0.011** (0.005)	0.175 (0.148)	−0.040 (0.112)
year5	−0.006 (0.061)	0.024*** (0.006)	0.188 (0.152)	0.102 (0.126)
year6	−0.014 (0.062)	0.080*** (0.023)	0.305* (0.158)	0.300** (0.140)
year7	−0.021 (0.060)	0.339*** (0.067)	−0.099 (0.148)	0.276* (0.147)
year8	−0.026 (0.059)	0.174*** (0.028)	0.082 (0.142)	0.066 (0.126)
year9	−0.038 (0.057)	0.210*** (0.054)	0.020 (0.149)	0.217 (0.138)
year10	−0.066 (0.057)	0.189*** (0.040)	−0.241* (0.142)	−0.159 (0.111)
zonaReserva:year1	−0.058 (0.078)	0.008 (0.006)	0.189 (0.232)	0.134 (0.144)
zonaReserva:year2	0.126 (0.121)	−0.0005 (0.007)	0.195 (0.256)	0.086 (0.177)
zonaReserva:year3	0.053 (0.093)	−0.008 (0.011)	−0.106 (0.211)	−0.072 (0.178)
zonaReserva:year4	0.035 (0.065)	0.024*** (0.009)	−0.140 (0.211)	0.448*** (0.167)
zonaReserva:year5	−0.045 (0.068)	−0.009 (0.009)	−0.233 (0.204)	−0.078 (0.169)
zonaReserva:year6	0.096 (0.075)	−0.042* (0.024)	−0.222 (0.223)	0.038 (0.169)
zonaReserva:year7	−0.030 (0.065)	−0.124 (0.089)	−0.080 (0.194)	−0.047 (0.199)
zonaReserva:year8	0.012 (0.069)	0.084 (0.082)	−0.082 (0.179)	0.120 (0.160)
zonaReserva:year9	0.002 (0.064)	0.036 (0.069)	−0.380** (0.181)	0.210 (0.181)
zonaReserva:year10	−0.016 (0.062)	−0.056 (0.065)	−0.189 (0.178)	0.167 (0.143)
Constant	0.122** (0.054)	0.016*** (0.003)	0.659*** (0.128)	0.567*** (0.094)
Observations	659	641	659	641
R^2	0.049	0.267	0.114	0.080
Residual Std. Error	0.189 (df = 637)	0.175 (df = 619)	0.526 (df = 637)	0.525 (df = 619)
Note:				*p<0.1; **p<0.05; ***p<0.01

Table E. Coefficient estimates of biological indicators for Maria Elena.

	Dependent variable:			
	Lobster density	Fish biomass	Invertebrate density	Fish density
	(1)	(2)	(3)	(4)
zonaReserva	-0.033^{**} (0.013)	-0.017 (0.027)	-0.008 (0.015)	-0.362 (0.758)
year1	0.008 (0.023)	0.075^{**} (0.029)	0.033^{*} (0.018)	0.028 (0.755)
year2	-0.019 (0.015)	0.034 (0.033)	-0.010 (0.015)	-0.046 (0.759)
year3	-0.028^{**} (0.014)	0.006 (0.027)	-0.017 (0.013)	-0.566 (0.700)
year4	0.023 (0.028)	0.031 (0.033)	0.044^{*} (0.026)	0.993 (0.952)
zonaReserva:year1	-0.003 (0.024)	0.064 (0.042)	-0.003 (0.023)	1.342 (0.915)
zonaReserva:year2	0.046^{*} (0.024)	0.017 (0.036)	0.035 (0.023)	0.181 (0.838)
zonaReserva:year3	0.074^{***} (0.024)	0.064 (0.070)	0.092^{*} (0.049)	0.139 (0.774)
zonaReserva:year4	0.001 (0.031)	0.003 (0.037)	-0.009 (0.029)	-0.953 (1.043)
Constant	0.033^{**} (0.013)	0.041 (0.025)	0.033^{**} (0.013)	1.462^{**} (0.691)
Observations	48	89	48	89
R^2	0.222	0.171	0.308	0.258
Residual Std. Error	0.038 (df = 38)	0.086 (df = 79)	0.043 (df = 38)	1.074 (df = 79)

Note: *p<0.1; **p<0.05; ***p<0.01

Table F. Coefficient estimates of biological indicators for Punta Herrero.

	Dependent variable:			
	Lobster density	Fish biomass	Invertebrate density	Fish density
	(1)	(2)	(3)	(4)
zonaReserva	0.004 (0.009)	0.212** (0.097)	0.192* (0.103)	2.570** (1.254)
year-1	−0.006 (0.007)	−0.045*** (0.016)	−0.018 (0.033)	−1.398*** (0.279)
year1	−0.008 (0.005)	−0.033** (0.016)	−0.053*** (0.013)	−1.057*** (0.279)
year2	0.004 (0.009)	−0.040*** (0.015)	−0.004 (0.026)	−1.112*** (0.312)
year3	−0.001 (0.006)	−0.031 (0.023)	0.025 (0.033)	−0.691 (0.498)
zonaReserva:year-1	0.010 (0.015)	0.264 (0.186)	−0.066 (0.121)	0.496 (1.312)
zonaReserva:year1	0.018 (0.024)	−0.148 (0.100)	0.041 (0.143)	−1.015 (1.348)
zonaReserva:year2	0.019 (0.021)	−0.044 (0.121)	−0.153 (0.110)	0.018 (1.561)
zonaReserva:year3	0.016 (0.016)	−0.167 (0.106)	−0.034 (0.134)	−1.575 (1.444)
Constant	0.011** (0.005)	0.060*** (0.015)	0.079*** (0.012)	2.009*** (0.259)
Observations	129	167	129	167
R^2	0.050	0.211	0.113	0.169
Residual Std. Error	0.048 (df = 119)	0.213 (df = 157)	0.263 (df = 119)	2.769 (df = 157)
Note:				*p<0.1; **p<0.05; ***p<0.01

Table G. Coefficient estimates of socioeconomic indicators in Isla Natividad.

	Dependent variable:	
	Landings	valor Revenues
	(1)	(2)
zonaReserva	−66.248*** (21.192)	−5.040* (2.817)
year-6	−9.747 (26.691)	6.074 (4.376)
year-5	−26.548 (36.201)	2.106 (7.819)
year-4	−8.805 (54.742)	−1.665 (5.141)
year-3	29.433 (53.606)	2.834 (5.409)
year-2	−17.752 (37.964)	3.423 (3.086)
year-1	−5.421 (46.563)	1.095 (3.894)
year1	−46.722* (24.836)	−1.873 (3.882)
year2	9.037 (29.383)	10.802** (5.477)
year3	−23.798 (21.935)	9.425* (5.316)
year4	−54.097** (24.541)	2.816 (5.541)
year5	48.012** (21.950)	10.380 (7.267)
year6	39.160 (39.516)	12.549 (7.653)
year7	−11.992 (30.520)	10.988 (12.703)
year8	−26.265 (37.453)	14.186 (14.328)
zonaReserva:year-6	−12.067 (26.691)	−1.980 (4.376)
zonaReserva:year-5	6.214 (36.201)	−1.715 (7.819)
zonaReserva:year-4	−26.453 (54.742)	−1.237 (5.141)
zonaReserva:year-3	−18.071 (53.606)	−2.213 (5.409)
zonaReserva:year-2	5.569 (37.964)	−2.142 (3.086)
zonaReserva:year-1	−13.914 (46.563)	−1.703 (3.894)
zonaReserva:year1	−0.696 (24.836)	−1.119 (3.882)
zonaReserva:year2	1.436 (29.383)	8.758 (5.477)
zonaReserva:year3	10.607 (21.935)	12.639** (5.316)
zonaReserva:year4	−6.110 (24.541)	2.515 (5.541)
zonaReserva:year5	−61.368*** (21.950)	0.047 (7.267)
zonaReserva:year6	−2.498 (39.516)	12.371 (7.653)
zonaReserva:year7	6.842 (30.520)	3.441 (12.703)
zonaReserva:year8	65.875* (37.453)	10.347 (14.328)
Constant	202.868*** (21.192)	15.517*** (2.817)
Observations	60	60
R^2	0.638	0.492
Residual Std. Error (df = 30)	43.483	9.794
Note:		*p<0.1; **p<0.05; ***p<0.01

Table H. Coefficient estimates of socioeconomic indicators in Maria Elena.

	Dependent variable:	
	Landings	valor Revenues
	(1)	(2)
zonaReserva	−12.452 (21.012)	−1.831 (4.026)
year-6	−10.495 (39.993)	−3.715 (5.741)
year-5	−1.865 (49.084)	−0.997 (8.401)
year-4	−7.648 (43.503)	1.206 (10.941)
year-2	2.857 (49.422)	−0.051 (8.558)
year-1	7.519 (40.701)	0.151 (6.850)
year1	−9.668 (21.613)	−1.990 (4.118)
zonaReserva:year-6	−7.993 (39.993)	−0.607 (5.741)
zonaReserva:year-5	−12.027 (49.084)	−2.239 (8.401)
zonaReserva:year-4	−11.520 (43.503)	−4.595 (10.941)
zonaReserva:year-2	−18.129 (49.422)	−3.360 (8.558)
zonaReserva:year-1	−11.395 (40.701)	−2.079 (6.850)
zonaReserva:year1	−9.629 (21.613)	−2.092 (4.118)
Constant	38.859* (21.012)	7.125* (4.026)
Observations	21	21
R^2	0.230	0.242
Residual Std. Error (df = 7)	39.665	7.294
Note:		*p<0.1; **p<0.05; ***p<0.01

Table I. Invertebrate species checklist.

Species	Isla Natividad	Maria Elena	Punta Herrero
Anthopleura spp	1	0	0
Cancer spp	1	0	0
Centrostephanus coronatus	1	0	0
Crassedoma giganteum	1	0	0
Cypraea spp	1	0	0
Diadema antillarum	0	1	1
Eucidaris tribuloides	0	1	1
Haliotis corrugata	1	0	0
Haliotis fulgens	1	0	0
Haliotis rufescens	1	0	0
Holothuroidea spp	0	1	1
Kelletia kelletii	1	0	0
Loxorhynchus grandis	1	0	0
Megastraea turbanica	1	0	0
Megastraea undosa	1	0	0
Megathura crenulata	1	0	0
Mesocentrotus franciscanus	1	0	0
Neobernaya spadicea	1	0	0
Octopus spp	1	0	1
Other sea urchins	0	1	1
Panulirus argus	0	1	1
Panulirus guttatus	0	1	1
Panulirus interruptus	1	0	0
Parastichopus parvimensis	1	0	0
Patiria miniata	1	0	0
Pisaster giganteus	1	0	0
Pycnopodia heliantoides	1	0	0
Stenopus hispidus	0	1	1
Strombus gigas	0	1	1
Strongylocentrotus purpuratus	1	0	0
Tripneustes esculentus	0	1	1

Table J. Fish species checklist.

Species	Isla Natividad	Maria Elena	Punta Herrero
Abudefduf saxatilis	0	1	1
Acanthurus	0	1	1
Aetobatus narinari	0	1	0
Anisotremus davidsonii	1	0	0
Anisotremus surinamensis	0	1	1
Anisotremus virginicus	0	1	1
Balistes vetula	0	1	1
Bodianus rufus	0	1	1
Calamus pennatula	0	1	1
Canthidermis sufflamen	0	0	1
Caranx bartholomaei	0	1	0
Caranx crysos	0	0	1
Caranx latus	0	1	0
Caranx ruber	0	1	1
Caulolatilus princeps	1	0	0
Cephalopholis cruentata	0	1	1
Cephalopholis fulva	0	1	1
Chaetodon	0	1	1
Chromis punctipinnis	1	0	0
Chromis spp	0	1	1
Embiotoca jacksoni	1	0	0
Epinephelus guttatus	0	0	1
Epinephelus striatus	0	1	0
Gerres cinereus	0	0	1
Girella nigricans	1	0	0
Haemulon aurolineatum	0	1	1
Haemulon carbonarium	0	1	1
Haemulon chrysargyreum	0	1	1
Haemulon flavolineatum	0	1	1
Haemulon macrostomum	0	1	0
Haemulon melanurum	0	0	1
Haemulon parra	0	1	1
Haemulon plumierii	0	1	1
Haemulon sciurus	0	1	1
Haemulon spp	0	0	1
Haemulon striatum	0	1	1
Halichoeres semicinctus	1	0	0
Heterodontus francisci	1	0	0
Holacanthus ciliaris	0	1	1
Holacanthus tricolor	0	1	1
Hypsypops rubicundus	1	0	0
Kyphosus spp	0	1	1
Lachnolaimus maximus	0	1	1
Lutjanus analis	0	1	1
Lutjanus apodus	0	1	1

Table J. Fish species checklist. *(continued)*

Species	Isla Natividad	Maria Elena	Punta Herrero
Lutjanus cyanopterus	0	0	1
Lutjanus griseus	0	1	1
Lutjanus jocu	0	1	1
Lutjanus mahogoni	0	1	1
Lutjanus spp	0	0	1
Lutjanus synagris	0	1	0
Melichthys niger	0	0	1
Microspathodon chrysurus	0	1	1
Mycteroperca bonaci	0	0	1
Ocyurus chrysurus	0	1	1
Ophiodon elongatus	1	0	0
Oxyjulis californica	1	0	0
Paralabrax clathratus	1	0	0
Paralabrax nebulifer	1	0	0
Pomacanthus arcuatus	0	1	1
Pomacanthus paru	0	1	1
Pterois volitans	0	1	1
Rhacochilus vacca	1	0	0
Rhinobatos productus	1	0	0
Scarus coeruleus	0	0	1
Scarus spp	0	1	1
Scarus vetula	0	1	0
Scomberomorus regalis	0	1	1
Scomberomorus spp	0	0	1
Scorpaenichthys marmoratus	1	0	0
Sebastes spp	1	0	0
Semicossyphus pulcher	1	0	0
Sparisoma aurofrenatum	0	1	1
Sparisoma viride	0	1	1
Sphyraena barracuda	0	0	1
Squatina californica	1	0	0
Stegastes spp	0	1	1
Stereolepis gigas	1	0	0
Thalassoma bifasciatum	0	1	1
